# Effect of Particle Velocity on Microcutting Process of Fe–C Alloy by Molecular Dynamics

**DOI:** 10.3390/mi13081339

**Published:** 2022-08-18

**Authors:** Chunxia Deng, Junye Li, Wenqing Meng, Weihong Zhao

**Affiliations:** 1 Ministry of Education Key Laboratory for Cross-Scale Micro and Nano Manufacturing, Changchun University of Science and Technology, Changchun 130022, China; 2 Chongqing Research Institute, Changchun University of Science and Technology, Chongqing 401135, China

**Keywords:** particle microcutting, molecular dynamics, Fe–C alloy, removal mechanism

## Abstract

In order to study the material removal mechanism of Fe–C alloy surfaces in the particle microcutting process, the molecular dynamics method was used to study the material deformation and removal rules during the particle microcutting process. By analyzing and discussing the particle cutting force, atomic energy, atomic displacement, lattice structure, and dislocation in the particle microcutting process under different cutting velocities, the material removal mechanism is revealed. The results show that the atomic binding energy of Fe–C alloy increases with an increase in particle cutting velocity. The cutting force of particles and atomic potential energy of the workpiece increase obviously. The accumulated strain energy and dislocation energy in the lattice increase, the lattice deformation becomes more severe, and the material is prone to plastic deformation. The atoms form atomic groups at the front of the particle and are then remove from the surface of Fe–C alloy in the form of chips.

## 1. Introduction

With the rapid development of science and technology, the precision requirements of mechanical parts are increasing day by day, among which abrasive flow machining (AFM) technology has been widely used in mechanical-related fields [1]. In the AFM process, particles distributed uniformly in the fluid medium can be regarded as countless tiny cutting tools. Under the repeated joint action of these cutting tools, deburring and grinding of the corners are realized, so that the workpiece surface can achieve the purpose of precision finishing [2].

In the AFM process, the impact cutting of particles on the workpiece is the main mechanism of surface finishing [3,4]. Material removal and wear properties have a significant impact on the application of the material [5]. The microcutting action of particles on the workpiece is a dynamic process occurring in the local area of the workpiece surface, and the material removal in the cutting area is a microscale phenomenon, so many macroscopic theories are not suitable for research on the mechanism of microcutting [6]. The material deformation and removal at the microscale are quite different from that of macroscopic cutting. At the microscale, the interaction between particle and materials is directly expressed at the atomic scale. Material removal is closely related to atomic interaction and atomic arrangement. The internal structure of the workpiece has an important effect on the deformation of the material. In order to study the characteristics of AFM microcutting, the molecular dynamics (MD) method is an effective method used to analyze the material changes in the local cutting area during the machining process at the microscale [7]. The MD method is a numerical simulation method based on Newtonian mechanics. The method generates a great deal of information about the structure, dynamics, and thermodynamics of microscopic particles by simulating the physical interactions between their atomic components [8]. The MD method is widely used in molecular-scale research to analyze molecular configuration and order, as well as thermodynamic and kinetic properties of systems [9,10].

Many achievements have been made in the simulation of ultra-precision grinding processes by MD. Choong et al. proposed a method for forming brittle materials by mixing deposition and mechanical cutting, and carried out large-scale simulations using MD to study surface and subsurface defects and morphologies [11]. Ranjan et al. used MD to study the mechanical polishing process of diamond particle on stainless steel, and proposed an economical and efficient polishing strategy [12]. Ren et al. studied the ultra-high-velocity grinding characteristics of single-crystal nickel through MD, and revealed the formation mechanism of chips and the relationship between the defects on the processed surface and the tangential indirection [13]. Papanikolaou et al. used MD to study the effect of rigidity on cutting depth and grinding process characteristics when a single abrasive particle is rubbed against the workpiece [14]. Markopoulos et al. simulated the cutting effect of diamond abrasive particles on a single-crystal copper workpiece, and studied the important role of abrasive morphology and orientation on chip formation, cutting force, and temperature changes during grinding [15]. Liu et al. used MD to simulate the material removal behavior and subsurface damage of single-crystal silicon during cutting, and observed the extrusion and shear transition phenomena of the material during cutting and the inhibition of cracking by temperature [16]. Fang et al. used MD to simulate the tribological properties of bilayer Cu/Ag materials and obtained the effects of tool machining parameters on material removal and subsurface damage [17]. In our previous work, we also used MD to simulate the material internal change and cutting mechanism of SiC grinding Fe–C alloy at a 5° cutting angle, and revealed the damage mechanism and defect change of the material subsurface layer [18].

For the AFM process, current research is mainly focused on computational fluid dynamics (CFD), which is a macroscopic study. For molecular and atomic levels, such as the change of workpiece surface morphology and inherent defects, the current research is still insufficient. In our previous paper [18], we only studied a single machining process at a speed of 60 m/s and cutting at 5°. However, speed plays a crucial role in the internal changes of the material during processing [7]. Therefore, this paper takes the velocity as the research variable and focuses on the influence of the velocity change on material internal defects and the cutting mechanism. In order to study the deformation and removal phenomena of Fe–C alloy workpieces at the microscale, MD simulation was carried out for an Fe–C alloy workpiece in particle microcutting. By analyzing the thermodynamic properties of the simulation system and the changes of the surface morphology and internal structure of the workpiece in the process of microcutting, the microcutting process of particle was studied from the microscale. The influence of different cutting velocities on AFM was discussed to reveal the mechanism of particles acting on the workpiece. This study provides a theoretical basis and technical support for the further study of AFM technology, which has certain academic significance and application value.

## 2. Model Parameters and Simulation Methods

Different particle cutting velocities will lead to different qualities of Fe–C alloy workpieces after particle microcutting. In order to investigate the influence of particle cutting velocity on the microcutting of Fe–C alloy, a simulation model of SiC particles cutting an Fe–C alloy workpiece is constructed.

Combined with the actual AFM process, the working environment is usually at room temperature. Although the grinding process causes heat due to friction, the heat will be carried away by the particles behind it. In this simulation, the initial temperature is set to 310 K, and the thermostat layer is set to take away the heat generated by the Newtonian layer, so as to meet the actual processing situation. According to the Fe–C phase diagram, Fe–C alloy is a combination of α-Fe and cementite Fe_3_C at room temperature. In this model, the carbon content in iron is 0.40%, and the body-centered cubic (BCC) lattice structure is the same as that of α-Fe. As for particle selection, we chose SiC material in a diamond cubic lattice and in the shape of a sphere with a radius of 15 Å.

**Figure 1:**
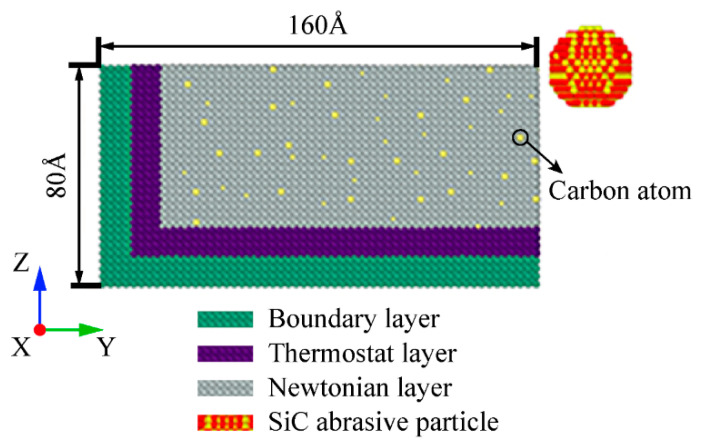
Microcutting simulation model.

As shown in Figure 1, the size of the model in X, Y, and Z directions is 100 Å × 160 Å × 80 Å, which contains 114,388 Fe atoms and 2144 C atoms, and the mass fraction of C atoms is 0.40%. The workpiece is divided into boundary layer, thermostat layer (used to transfer heat), and Newtonian layer (to simulate the motion of atoms). Periodic boundary conditions are used in the *X*-axis direction of the Fe–C alloy workpiece to reduce the size effect [19]. The initial temperature of the system is set at 310 K, and the system is relaxed for 10,000 steps by using the Nose–Hoover hot-bath method under the canonical ensemble (NVT) to achieve steady state. The SiC particles are initially placed on the upper right of the Fe–C alloy workpiece, 5 Å away from its edge, to avoid interaction between them. Grinding is simulated by the micro-canonical ensemble (NVE). SiC particles are cut along the negative direction the of *Y*-axis at different cutting velocities of 40 m/s, 50 m/s, 60 m/s, 70 m/s, 80 m/s, and 90 m/s, respectively. The initial cutting depth of potential function plays a decisive role in the simulation results. In this simulation, it is set at 15 Å; the number of steps for numerical analysis is 140,000, and the time step is 1 fs.

The interaction between atoms controls the interaction behavior between atoms and fundamentally determines all properties of materials. This interaction is described by the potential function. In our MD simulation, the selection study, the embedded-atom method (EAM) potential is selected to describe the interaction between Fe–Fe, Fe–C, and C–C in the Fe–C alloy workpiece [20,21]. This EAM potential function is widely used in molecular dynamics simulations of Fe–C alloys [22,23,24]. Morse potential is selected to describe the interaction between Fe–Si, Fe–C (SiC), C–C (SiC), and C–Si in the Fe–C alloy workpiece and SiC particles [25]. Tersoff potential is selected to describe the interaction between C–C, C–Si, and Si–Si in SiC particles [26]. In this paper, the MD simulation is conducted using Large-scale Atomic/Molecular Massively Parallel Simulator (LAMMPS) [27], and the simulation results are visualized using the Open Visualization Tool (OVITO) [28].

## 3. Results and Discussion

### 3.1. Effect of Particle Cutting Force on Surface Material Removal of Fe–C Alloy

The cutting force exerted by particles on the Fe–C alloy workpiece atoms can indirectly reflect the material removal rule and the cutting process, and it is an important physical parameter in the cutting action. In order to investigate the influence of particle cutting velocity on the cutting force variation, the particle cutting force variation under different velocity conditions in the microcutting process was studied.

**Figure 2:**
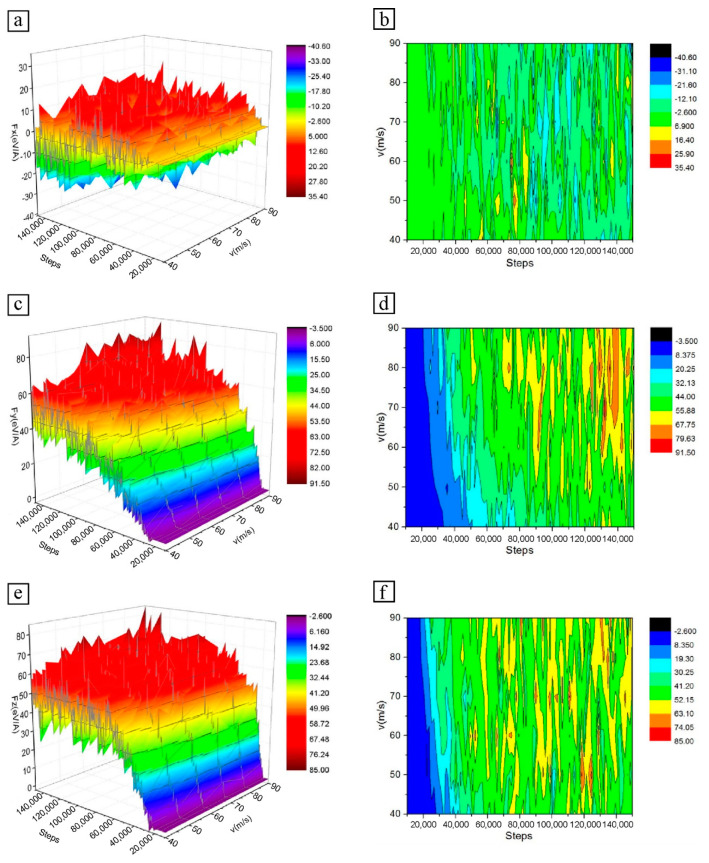
Variation of cutting force of SiC particles at different particle cutting velocities: (**a**) 3D curve diagram of cutting force in X direction; (**b**) Isoline diagram of cutting force in X direction; (**c**) 3D curve diagram of cutting force in Y direction; (**d**) Isoline diagram of cutting force in Y direction; (**e**) 3D curve diagram of cutting force in Z direction; (**f**) Isoline diagram of cutting force in Z direction.

Figure 2 shows the 3D curve diagram and isoline diagram of the cutting force changing with the number of simulated steps at different velocities. Since the cutting force of particles in the X direction comes from the friction between particle atoms and workpiece atoms, Fx only fluctuates up and down along Fx = 0 eV/Å in the cutting process, and its overall trend does not show a significant increase or decrease. The cutting forces Fy and Fz of particles in Y and Z directions gradually increase to a stable value and then continue to fluctuate within a certain range. According to the isoline diagram, with an increase in particle cutting velocity, the cutting forces in both Y and Z directions increase, among which the main cutting force Fy is the most obvious.

At the initial stage of microcutting, the SiC particles and the Fe–C alloy workpiece have not yet made contact, and the cutting force of the particles is a collection of repulsion forces between atoms. At this time, the cutting velocity of particles has no direct influence on the cutting force, so the cutting force is the same at different velocities. However, the cutting distance is different at the same time due to different cutting velocities. With the progress of microcutting, the high-velocity particles make contact with the atoms of the workpiece. Before the initial deformation of the workpiece can occur, the particle atoms need to overcome the bond energy between the Fe–C alloy atoms; that is, to break the chemical bond between Fe atoms and C atoms, and force the workpiece atoms to produce a large displacement. This results in the increase of the cutting force of SiC particles.

When the low-velocity particles come into contact with the workpiece, the high-velocity particles have completely cut into the surface of the workpiece, and the number of atoms that have made contact with the particles increases. By the combined effects of surface hardening, the corresponding inter-atomic bonding forces gradually increase and the bonding energy needed to be overcome becomes larger. As the particles move, the atoms of the workpiece in the cutting area accumulate in front of the particles, which has a great effect on the cutting of particles. As a result, the cutting force gradually increases. When the low-velocity particles cut into the workpiece surface completely, the high-velocity particles have entered the stable cutting stage. The atom accumulation in the front end of the particles becomes larger, which further impedes the cutting. The cutting force of high-velocity particles increases due to the joint hindrance of atomic bond energy and atomic stacking.

In the late cutting period, the cutting states of particles with different velocities gradually converge. Although there are some differences, the overall cutting force change trend is consistent. In the process of microcutting, the cutting force fluctuates continuously. The main reason for this is that the energy generated by lattice deformation keeps accumulating and releasing, forcing lattice reconstruction.

### 3.2. Effect of Particle Cutting Energy on Surface Material Removal of Fe–C Alloy

#### 3.2.1. Kinetic Energy Analysis

In order to study the effect of kinetic energy on the surface material removal of Fe–C alloy in the microcutting process, the numerical analysis of the particle microcutting process under different velocity conditions is carried out, and the influence of atomic kinetic energy variation on the microcutting process of the Fe–C alloy workpiece is discussed.

**Figure 3:**
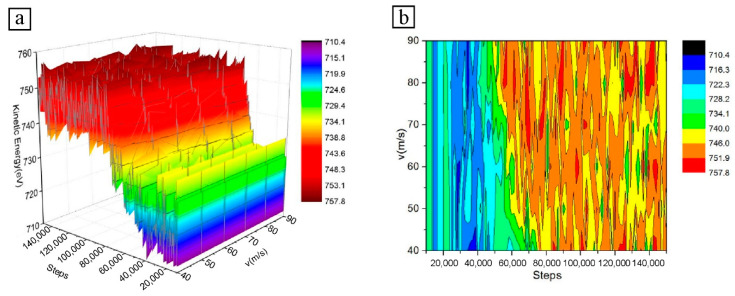
Atomic kinetic energy diagram at different particle velocities: (**a**) 3D curve diagram; (**b**) Isoline diagram.

The variation of atomic kinetic energy with the number of simulation steps for different particle velocity conditions is shown in Figure 3. The atomic kinetic energy of the Fe–C workpiece in the 3D curve diagram fluctuates during the microcutting process. This phenomenon arises mainly from the dynamic balance between the interconversion of atomic potential and kinetic energies. The generation of dislocations and the deformation of the lattice due to the motion also cause a continuous accumulation and release of strain energy.

In the early stages of cutting, the atomic kinetic energy of the workpiece increases with increasing cutting velocity. The reason for this is that the high-velocity particles cut longer distances in the same amount of time, and more atoms accumulate at the front of the particles. However, as the smallest-velocity particles cut completely into the workpiece surface, the atomic build-up occurs at the front of the particles and the difference in build-up is not significant. Therefore, the effect of different particle velocities on the atomic kinetic energy of the workpiece decreases in the later stages of microcutting.

In Figure 3, the change in cutting velocity does not cause a significant change in the atomic kinetic energy of the Fe–C workpiece, which varies very little. Although the microcutting effect of the particles on the material results in local Fe–C atoms having a certain velocity on the surface of the workpiece, some of the atoms in the cutting area are displaced a certain distance to either side of the particle and then accumulate on the surface. Some of the atoms move downwards and form a machined surface under friction; the velocity of these atoms does not change significantly depending on the particle velocity. Only a small number of workpiece atoms move with the particles at the same velocity at the front of the particles, where they gradually accumulate to a certain extent and are removed from the workpiece surface by the particles in the form of chips.

In short, a smaller proportion of the workpiece atoms are displaced and an even smaller proportion move forward with the particles at the same velocity and are finally discharged as chips. Therefore, the particle cutting velocity does not have much influence on the displacement velocity of the workpiece atoms.

Temperature has the greatest influence on the kinetic energy of the atoms of the Fe–C workpiece in the simulation system. In NVT, most of the heat generated during cutting is transferred to the thermostat layer, where the temperature difference in the local atoms caused by the different cutting velocities of the particle also has only a small effect on the kinetic energy. Therefore, the different velocity conditions can only cause a weak change in the total kinetic energy of the workpiece atoms.

#### 3.2.2. Potential Energy Analysis

In order to investigate the effect of particle velocity on atomic potential energy of the workpiece, the variation of atomic potential energy under different particle velocities during microcutting is studied.

**Figure 4:**
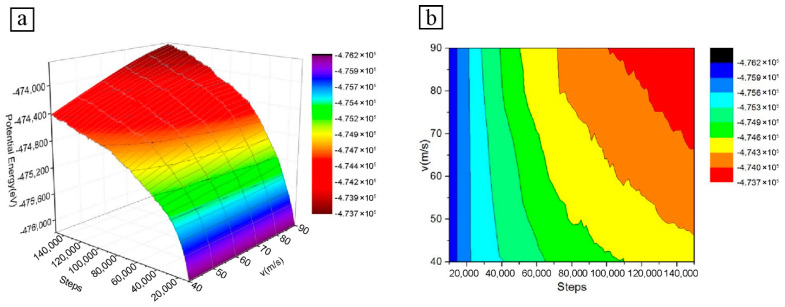
Atomic potential energy diagram at different particle velocities: (**a**) 3D curve diagram; (**b**) Isoline diagram.

The variation of atomic potential energy at different velocities is shown in Figure 4. The atomic potential energy of the workpiece increases with ab increase in cutting velocity. At the initial stage of particle microcutting, the initial cutting force is approximately equal to the sum of the repulsive forces between SiC particle atoms and Fe–C alloy atoms. The atomic repulsion force causes the atoms of the workpiece close to the particle to be squeezed, and a small amount of the atoms of the Fe–C alloy workpiece appear to achieve a small level of displacement. The local lattice structure deforms slightly, the atomic potential energy increases, and the resulting strain energy accumulates continuously in the lattice. In this stage, there is not much difference between values of atomic potential energy at different particle velocities.

As the microcutting progresses, the particles with higher velocity are already in contact with the surface of the Fe–C workpiece. The displacement of Fe and C atoms in the Fe–C workpiece gradually increases under the action of cutting force, resulting in a higher atomic potential energy. The relatively small-velocity particles are still not in contact with the Fe–C workpiece, and there is no obvious atomic potential energy change

By the time the low-velocity particles come into contact with the Fe–C workpiece, the high-velocity particles have already cut completely into the workpiece. In contrast, not only is the displacement of the atoms of the high-velocity particles greater, but also more strain energy and dislocation energy from lattice deformation is released during the microcutting process. By the time the low-velocity particles have completely cut into the workpiece, the high-velocity particles have already cut a long distance on the workpiece surface, driving more atomic movement and relatively large displacement in the cutting area. The lattice and dislocation changes are more intense, and the strain energy accumulated in the lattice is released and transformed into atomic potential energy. As the particle velocity increases, the atomic potential energy of the workpiece increases significantly at this stage.

#### 3.2.3. Total Energy Analysis

**Figure 5:**
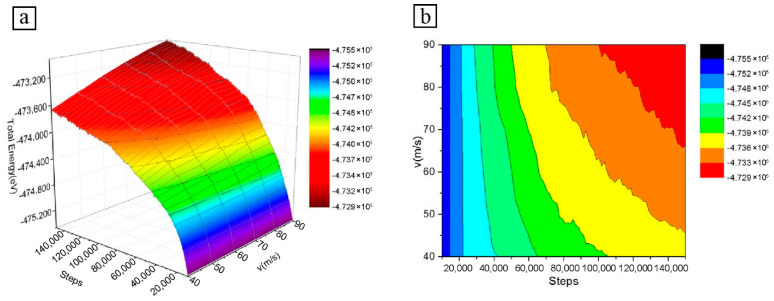
Atomic total energy diagram at different particle velocities: (**a**) 3D curve diagram; (**b**) Isoline diagram.

Figure 5 shows the variation of the total energy with the number of simulation steps for different particle cutting velocities. The total thermodynamic energy is the sum of the kinetic and potential energy of the atoms. From Figure 3 and Figure 4, the atomic kinetic energy is low in relation to the potential energy during microcutting. A comparison of Figure 4 and Figure 5 shows that the change in total energy follows a very similar trend to the change in potential energy. As the cutting velocity increases, the total atomic energy of the Fe–C workpiece also tends to increase gradually, as does the atomic potential energy.

The reason for this is that the effect of cutting velocity on the kinetic energy of the atoms is not as significant as the potential energy of the atoms, and that the kinetic energy is a smaller proportion of the total energy, with the difference between the total atomic energies arising mainly from the effect of different velocities on the potential energy of the atoms. The different cutting velocities of the particles also result in different cutting distances on the surface of the Fe–C workpiece and, therefore, corresponding differences in workpiece atomic displacement and local atomic velocities, which affect the lattice deformation and dislocation, change within the material. The combination of the various changes in the workpiece caused by the different cutting velocities manifests itself as a difference in the total atomic energy.

### 3.3. Effect of Particle Cutting Atom Displacement on Surface Material Removal of Fe–C Alloy

In order to investigate the influence of particle microcutting velocity on the atomic displacement of the Fe–C alloy workpiece surface, and to discuss the action form of particles on the workpiece, the material removal process, chip generation, and change under different microcutting velocity conditions, different atomic displacements in the workpiece are marked with different colors.

**Figure 6:**
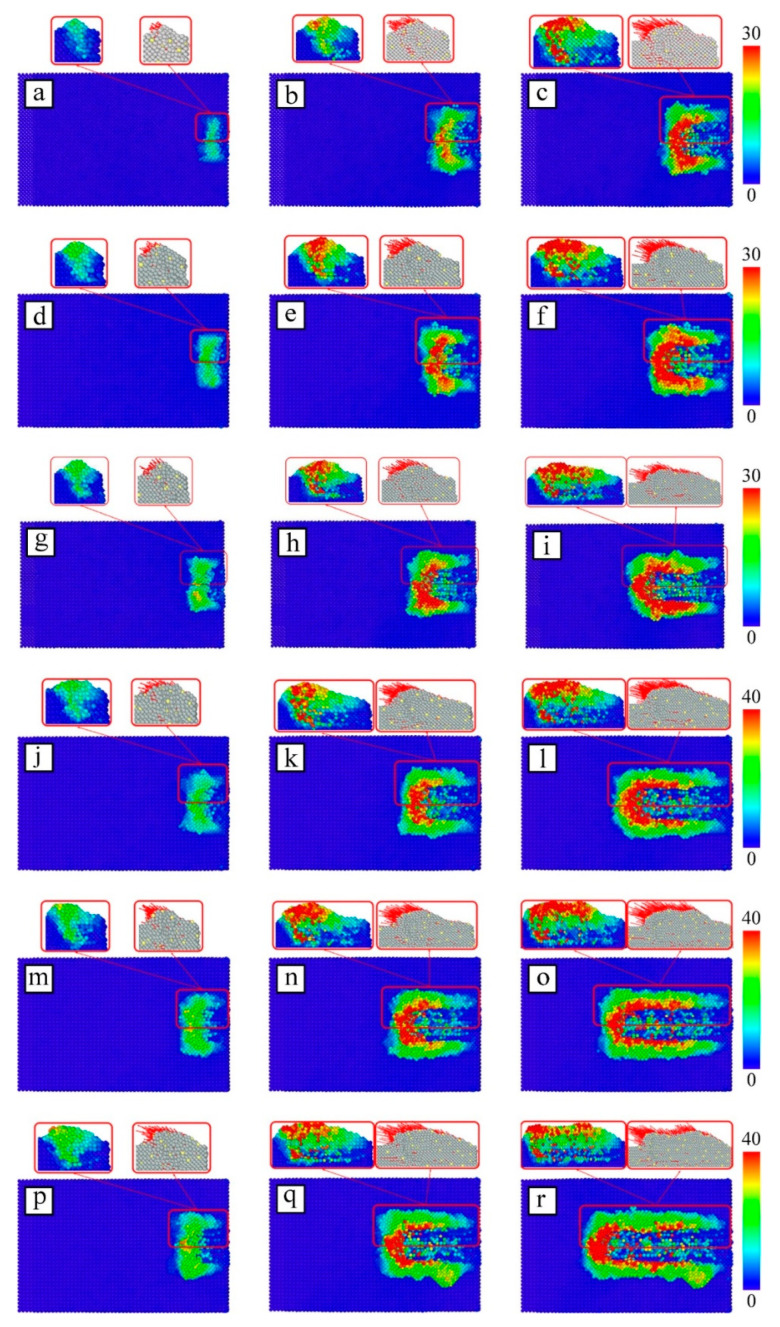
Atomic displacement diagram of workpiece at different particle velocities: (**a**) v = 40 m/s, t = 40 ps; (**b**) v = 40 m/s, t = 80 ps; (**c**) v = 40 m/s, t = 120 ps; (**d**) v = 50 m/s, t = 40 ps; (**e**) v = 50 m/s, t = 80 ps; (**f**) v = 50 m/s, t = 120 ps; (**g**) v = 60 m/s, t = 40 ps; (**h**) v = 60 m/s, t = 80 ps; (**i**) v = 60 m/s, t = 120 ps; (**j**) v = 70 m/s, t = 40 ps; (**k**) v = 70 m/s, t = 80 ps; (**l**) v = 70 m/s, t = 120 ps; (**m**) v = 80 m/s, t = 40 ps; (**n**) v = 80 m/s, t = 80 ps; (**o**) v = 80 m/s, t = 120 ps; (**p**) v = 90 m/s, t = 40 ps; (**q**) v = 90 m/s, t = 80 ps; (**r**) v = 90 m/s, t = 120 ps.

Figure 6 shows the displacement of atoms on the surface at different particle cutting velocities of 40 ps, 80 ps, and 120 ps. The arrows on the atoms in the partial profile indicate the displacement vector. The higher cutting velocity makes the cutting distance of SiC particles on the workpiece surface become longer; the number of workpiece atoms that produce displacement increases and the displacement of some atoms increases, and the local deformation of the material in the cutting area intensifies. As can be seen, the cutting velocity has a direct influence on the forming time of the machined surface. The higher the velocity, the faster the surface of the workpiece is formed.

Now, we contrast the changes in atomic profile and displacement of the Fe–C workpiece at different cutting velocities. The high-velocity particles are the first to cut into the Fe–C workpiece, causing plastic deformation of the material surface. The low-velocity particles are later in making contact with the workpiece, and only the initial elastic deformation exists. As the microcutting proceeds, the high-velocity particles not only cut longer distances on the surface of the Fe–C workpiece, but also squeeze some of the atoms to the sides and below the particle, while also pushing a small number of atoms at the front of the particles at the same velocity. When these atoms have accumulated to a certain extent at the front of the particle, they are stripped from the surface of the workpiece in the form of chips.

### 3.4. Analysis of Lattice Structure Change of Workpiece

Ackland–Jones bond angle analysis [29] is used to investigate the changes in the lattice structure within the Fe–C workpiece during particle microcutting with cutting velocity as a variable, and to investigate the atomic arrangement of the workpiece’s Newtonian layer. The type and distribution of defective structures within the material can be identified based on the calculated crystal structure type.

**Figure 7:**
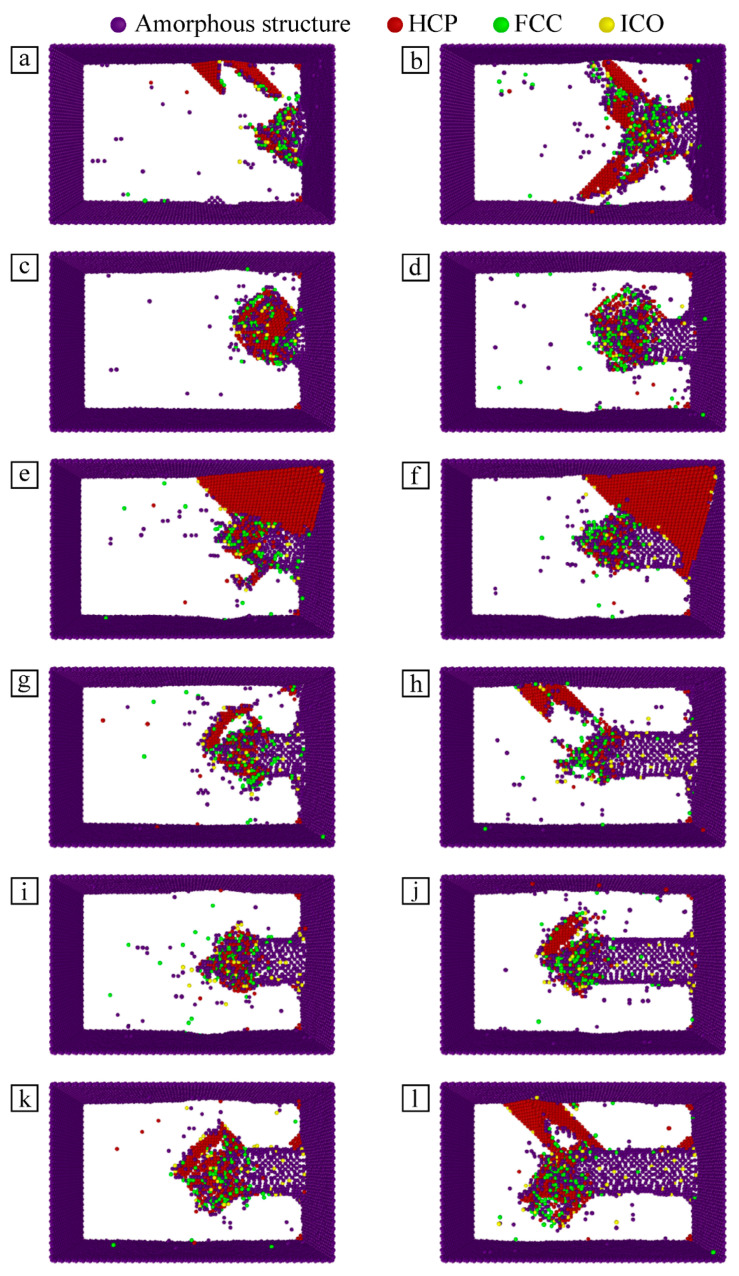
Lattice structure diagram of workpiece cutting layer under different particle cutting velocities: (**a**) v = 40 m/s, t = 70 ps; (**b**) v = 40 m/s, t = 110 ps; (**c**) v = 50 m/s, t = 70 ps; (**d**) v = 50 m/s, t = 110 ps; (**e**) v = 60 m/s, t = 70 ps; (**f**) v = 60 m/s, t = 110 ps; (**g**) v = 70 m/s, t = 70 ps; (**h**) v = 70 m/s, t = 110 ps; (**i**) v = 80 m/s, t = 70 ps; (**j**) v = 80 m/s, t = 110 ps; (**k**) v = 90 m/s, t = 70 ps; (**l**) v = 90 m/s, t = 110 ps.

Figure 7 shows the lattice structure diagram of the cutting zone section of the Fe–C alloy workpiece at different cutting velocities at the simulation times of 70 ps and 110 ps. Due to a certain degree of distortion of the lattice containing carbon atoms in the workpiece prior to microcutting, some of the atoms are identified as other cubic structures despite the small atomic offset. In order to facilitate the observation and analysis of structural changes in the cutting layer of the workpiece, such atoms of the workpiece that still maintain the original structure before microcutting are hidden in Figure 7. In the microcutting process of SiC particles, the Fe–C alloy material includes body-centered cubic (BCC) lattice structure, hexagonal close-packed (HCP) lattice structure, face-centered cubic (FCC) structure, icosahedron (ICO) structure, and amorphous structure.

In order to overcome the atomic bond energy before the material is removed, the cutting force must gradually rise to the extreme value beyond the binding force between atoms, so as to destroy the interaction between atoms. During this period, the particle cutting force disrupts the original lattice in the cutting region of the Fe–C alloy workpiece and destroys the orderly lattice structure inside the material. Bonds between atoms are broken and reborn, resulting in some atomic arrangements gradually becoming disordered and some amorphous structures in the cutting area. As the particle move, the number of amorphous structures gradually increases. Amorphous structures are basically distributed in the processed area. Some of the amorphous structures will be transformed into other lattice structures under the action of microcutting, and some of them will be removed in the form of chips, as described above.

With the progress of microcutting, the Fe–C alloy material is subjected to continuous friction. Some of the atomic layers slip on the dense plane of the crystal and result in dislocations. Part of original lattice gradually transforms into HCP structures. The local strain increases, forcing the atoms gradually out of equilibrium. When the strain variable breaks through the bearing limit before phase transformation, the local workpiece atoms become metastable. In a metastable state, HCP structures gradually increase in number, and some atomic stress is released. As a result, the temperature in the system has an increasing trend, which drives the growth of atomic kinetic energy. Under the joint action of shear force and friction force, the amorphous structure in the cutting area shifts with the continuous movement of particles, which changes the atomic arrangement state again, and the amorphous structure begins to change to HCP structure. At the same time, part of the HCP structures generated in the previous process gradually transform into amorphous structures.

According to the analysis of the atomic potential energy above, with the progress of microcutting, Fe atoms and C atoms are displaced under the action of cutting force, which destroys the crystal lattice structure. The elastic stress field is generated inside the material, which continuously stores the strain energy in the lattice. When the strain energy increases to the lattice bearing limit, the original lattice structure is disturbed to release the strain energy. The atoms of the workpiece are forced to rearrange in the form of low-energy lattice structures, and part of the BCC structure gradually transforms into an FCC structure with the movement of particles. When SiC particles cut and extrude the atoms, the original lattice structure is destroyed, and a small amount of ICO structure is also generated in the material. Compared with other crystal structures, FCC and ICO structures contribute relatively small percentages, so they have little influence on the microcutting process of particles.

By comparing the changes of the internal structure of the Fe–C alloy workpiece with different particle cutting velocities, it is found that there is always a variety of crystal structures in the material, and their quantity and distribution change obviously. However, there is no linear relationship between this change and particle cutting velocity. Different cutting velocities result in the change of atomic energy, which affects the accumulation and release of strain energy in the material. According to the changes in material cutting layers (in Figure 7a–l), the cutting distances of particles on the workpiece surface are different at different cutting velocities. At the same simulation time, the relative positions of particles and workpiece are different, so the stress states of atoms in the cutting area and the energy of local atomic groups are greatly different, and the lattice deformation degree is also significantly different.

### 3.5. Analysis of Dislocation Change in Workpiece

**Figure 8:**
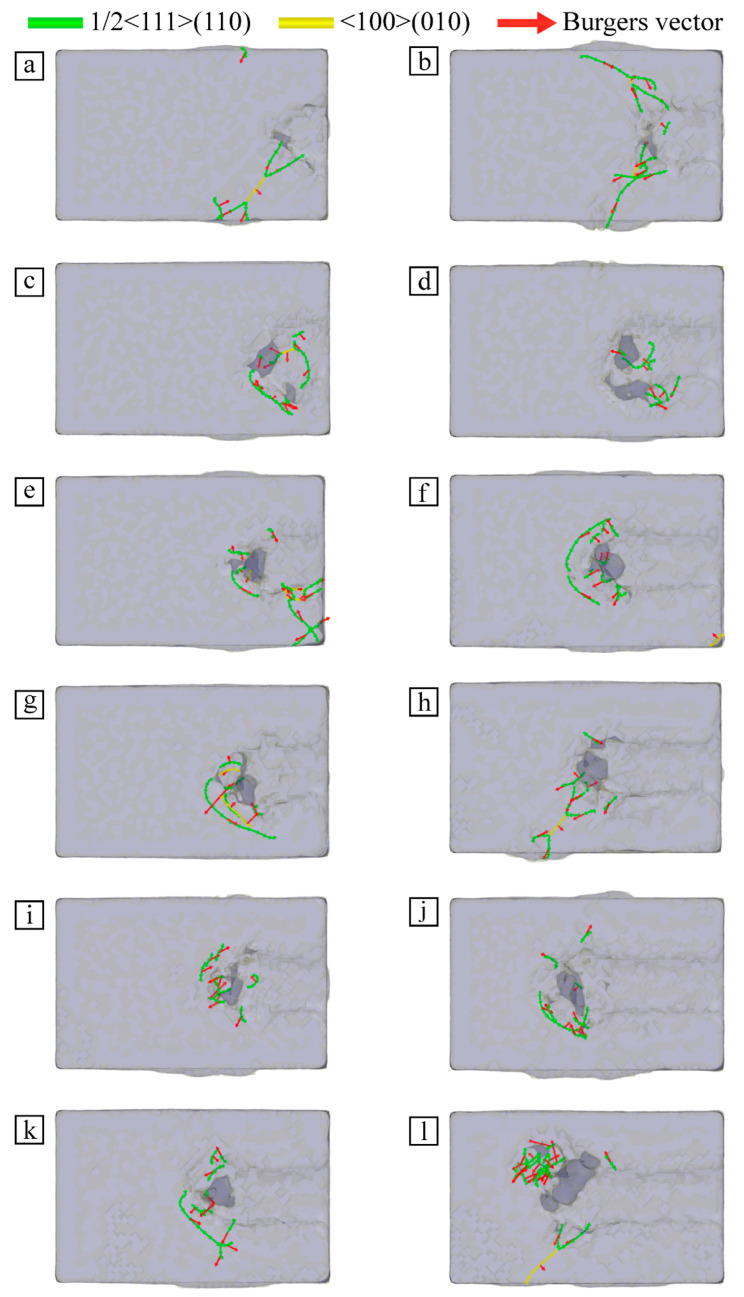
Internal dislocation distribution of workpiece at different velocities: (**a**) v = 40 m/s, t = 70 ps; (**b**) v = 40 m/s, t = 110 ps; (**c**) v = 50 m/s, t = 70 ps; (**d**) v = 50 m/s, t = 110 ps; (**e**) v = 60 m/s, t = 70 ps; (**f**) v = 60 m/s, t = 110 ps; (**g**) v = 70 m/s, t = 70 ps; (**h**) v = 70 m/s, t = 110 ps; (**i**) v = 80 m/s, t = 70 ps; (**j**) v = 80 m/s, t = 110 ps; (**k**) v = 90 m/s, t = 70 ps; (**l**) v = 90 m/s, t = 110 ps.

Under the combined action of cutting and friction of SiC particles, the atoms of the Fe–C alloy workpiece shift and change the original configuration. The atomic lattice structure in the cutting area is destroyed, and the local atoms demonstrate a regular dislocation phenomenon, which leads to dislocation in the workpiece. In order to discuss the change of internal dislocations in material at different cutting velocities, the discrete extraction algorithm (DXA) [30] is used to identify the dislocation defects in material, as shown in Figure 8.

Figure 8 shows the internal dislocation distribution of the Fe–C alloy workpiece at different velocities. The internal dislocation distribution of the workpiece at different cutting velocities is analyzed from the upper surface of the workpiece at t = 70 ps and 110 ps, respectively, and the Burgers vector is marked by a red arrow. It can be seen that there are ½ <111>(110) dislocations and <100>(010) dislocations in the Fe–C alloy workpiece at different cutting velocities. However, there were significant differences in the number and distribution of dislocations, and 1/2<111>(110) was the majority of the dislocations in the material. Due to the greater cutting velocity, the longer the cutting distance of SiC particles on the workpiece surface, the higher the number of displaced atoms in the workpiece, and some atoms move farther. This leads to aggravation of the local deformation of the material in the cutting area; this results in numbers and distributions of various dislocations that are significantly different.

According to the dislocation variation in Figure 8a–l, the faster the particle cutting velocity is, the more dislocations are found in the workpiece and the denser the cutting area. Most of the dislocations are concentrated near the grain and the lattice changes are complex. This phenomenon arises not only from the differences in atomic arrangement under microcutting action, but also from the trace increase in particle shear stress due to the increase in cutting speed. This leads to an enhanced inhomogeneous stress field and a small increase in the number of dislocations proliferating. At the same time, the change of atomic energy caused by the different cutting velocities also influences differences in the dislocation change degree in the material at different cutting velocities, which affects the accumulation and release of strain energy. Excessive particle cutting velocities exceed the threshold of the alloy’s ability to return to steady state, so dislocations are not transferred to adjacent locations. So, a large amount of strain energy blocks in the processing area, leaving a large number of dislocations.

## 4. Conclusions

In order to investigate the mechanism of particle cutting velocity on the removal of Fe–C alloy surface material, an MD model of Fe–C alloy surface material is established. This paper analyzes and discusses the surface action process of particle microcutting Fe–C alloy under the cutting velocities of 40 m/s to 90 m/s. The particle cutting force, microcutting energy, atomic displacement, lattice structure, and dislocation variation law in the particle microcutting process are studied, and the following conclusions are obtained:

(1) In the process of particle microcutting the Fe–C alloy workpiece, the main cutting force of particles increases with an increase in cutting velocity. The particles overcome the interatomic forces and bond energies of the Fe–C alloy, resulting in lattice reconstruction and amorphous structures, and the atomic accumulation of the material is removed in the form of chips;
(2) Under the cutting and friction action of particles, atomic kinetic energy and potential energy are transformed into each other, and energy maintains dynamic equilibrium. With an increase in particle cutting velocity, the atomic binding energy of Fe–C alloy increases, and the particle cutting force and atomic potential energy of workpiece increase obviously. The accumulated strain energy and dislocation energy in the lattice increase, causing lattice deformation and dislocation generation and change in the material;
(3) The cutting distance of particles on the surface of Fe–C alloy increases with an increase in particle cutting velocity. This makes the atomic displacement increase, the deformation of the material in the cutting area intensifies, and the material surface produces plastic deformation. Atoms gradually accumulate at the front of particles to form an atomic group, which is cut off from the workpiece surface by particles in the form of chips;
(4) With the process of particle microcutting, the atomic layer slips on the crystal close plane and dislocations occur in the Fe–C alloy. The structural transformation of the original lattice occurs, the structural mechanics quantity changes abruptly, the atomic kinetic energy increases, the crystal lattice structure is destroyed, and the elastic stress field is generated inside the material. As the atoms of the Fe–C alloy workpiece shift, the original configuration changes, and the lattice structure in the cutting area is destroyed. Atomic dislocations occur regularly, resulting in dislocations within the workpiece, and the surface material of Fe–C alloy is effectively removed.
